# Magnolol Reduces Glutamate-Induced Neuronal Excitotoxicity and Protects against Permanent Focal Cerebral Ischemia Up to 4 Hours

**DOI:** 10.1371/journal.pone.0039952

**Published:** 2012-07-09

**Authors:** Wei-Ting Lee, Miao-Hui Lin, E-Jian Lee, Yu-Chang Hung, Shih-Huang Tai, Hung-Yi Chen, Tsung-Ying Chen, Tian-Shung Wu

**Affiliations:** 1 Neurophysiology Laboratory, Neurosurgical Service, Departments of Surgery, National Cheng Kung University Hospital, College of Medicine, National Cheng Kung University, Tainan, Taiwan; 2 Institute of Biotechnology, National Cheng Kung University, Tainan, Taiwan; 3 Department of Early Childhood Education, National University of Tainan, Tainan, Taiwan; 4 Institute of Pharmacy, China Medical University, Taichung, Taiwan; 5 Department of Anesthesiology, Buddhist Tzu-Chi University and Buddhist Tzu Chi General Hospital, Hualien, Taiwan; 6 Institute of Chemistry, National Cheng Kung University, Tainan, Taiwan; Massachusetts General Hospital/Harvard Medical School, United States of America

## Abstract

Neuroprotective efficacy of magnolol, 5,5′-dially-2,2′-dihydroxydiphenyl, was investigated in a model of stroke and cultured neurons exposed to glutamate-induced excitotoxicity. Rats were subjected to permanent middle cerebral artery occlusion (pMCAO). Magnolol or vehicle was administered intraperitoneally, at 1 hr pre-insult or 1–6 hrs post-insult. Brain infarction was measured upon sacrifice. Relative to controls, animals pre-treated with magnolol (50–200 mg/kg) had significant infarct volume reductions by 30.9–37.8% and improved neurobehavioral outcomes (*P*<0.05, respectively). Delayed treatment with magnolol (100 mg/kg) also protected against ischemic brain damage and improved neurobehavioral scores, even when administered up to 4 hrs post-insult (*P*<0.05, respectively). Additionally, magnolol (0.1 µM) effectively attenuated the rises of intracellular Ca^2+^ levels, [Ca^2+^](i), in cultured neurons exposed to glutamate. Consequently, magnolol (0.1–1 µM) significantly attenuated glutamate-induced cytotoxicity and cell swelling (*P*<0.05). Thus, magnolol offers neuroprotection against permanent focal cerebral ischemia with a therapeutic window of 4 hrs. This neuroprotection may be, partly, mediated by its ability to limit the glutamate-induced excitotoxicity.

## Introduction

Ischemic stroke is characterized by overstimulation of glutamate receptors of the N-methyl-D-aspartate type (NMDARs) and increased inflow of intracellular Ca^2+^, [Ca^2+^](i) [1]–[5]. NMDAR overactivation disrupts antioxidant defenses and critical survival pathways, which not only increase the susceptibility of neurons and glia to ischemic damage but also trigger numerous ischemic cascades, leading to further neuronal degeneration, swelling, or even deaths [1], [2]. Nonetheless, efforts to inhibit NMDARs have generally failed, mainly due to critical roles of these receptors in neuronal survival and synaptic plasticity [2], [3]. Other strategies to improve neuronal defense against the glutamate-induced excitotoxicity and/or to decrease the [Ca^2+^](i) inflow into the ischemic neurons have, therefore, been suggested 4–7. Several different classes of antioxidants and/or neuroprotectants such as calpain inhibitors have been shown to protect against ischemia-induced excitotoxicity and, thus, decrease brain damage caused by experimental stroke [6]–[10].

Magnolol, a blood-brain barrier permeable phenolic constituent (5, 5′-dially-2, 2′-dihydroxydiphenyl) of magnolia bark, is known to be a central nervous system depressant agent and potent antioxidant [11]–[13]. Magnolol has been shown to protect against brain damage in an experimental heatstroke model [14]. We have previously shown that magnolol protects against hind limb ischemic-reperfusion injury in rats by reducing post-ischemic rises in the levels of nitrite/nitrate (NOX), malondialdehyde (MDA) and myeloperoxidase (MPO) [13]. Alternatively, magnolol is an inhibitor of voltage-dependent Ca^2+^ channel and can reduce necrotic cell deaths in mixed neuron-astrocyte cultures exposed to chemical hypoxia [15]–[17]. Accordingly, we suspected that magnolol might protect the brain against ischemic stroke.

In the study, we evaluated the protective effects of magnolol against cell damage and swelling as well as increased inflow of [Ca^2+^](i) in cultured neurons exposed to glutamate. Additionally, we investigated neuroprotective efficacy of magnolol in rats subjected to permanent focal cerebral ischemia.

**Figure 1:**
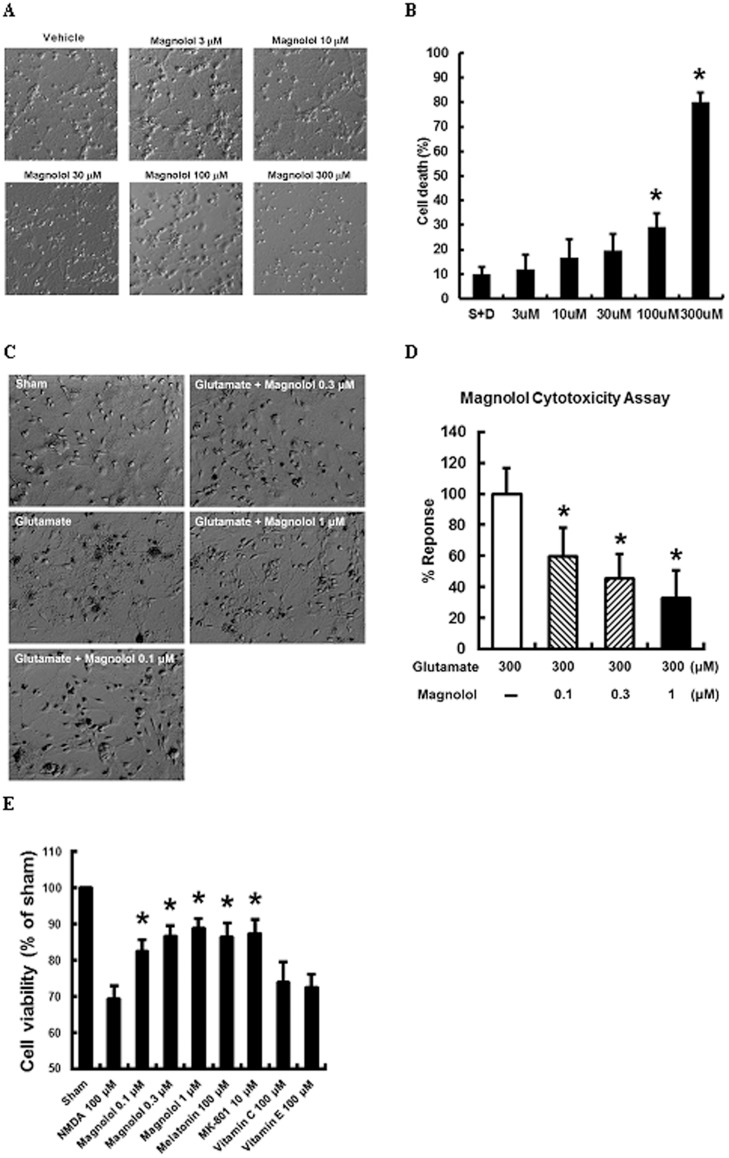
Magnolol reduced glutamate-and N-methyl-D-aspartate (NMDA)-induced cell deaths. (A, B) Differential interference contrast (DIC) photomicrographs showed neurotoxicity of magnolol at a concentration beyond 100 µM. (C, D) Magnolol (0.1–1 µM) achieved potent cytoprotection against glutamate-induced neuronal damage. (E) Magnolol (0.1–1 µM) achieved potent cytoprotection against NMDA-induced neuronal damage. *P<0.05 vs controls, and *n* = 5–9 per group.

## Results

Neurotoxicity of magnolol was observed with a concentration beyond 100 µM. The LD_50_ value was 129.3±0.1 µM (*P<*0.05; Figs. 1A, B). Alternatively, the glutamate- and NMDA-induced neurotoxicity was significantly attenuated by magnolol at 0.1–1.0 µM (*P<*0.05; Figs. 1C–E, respectively), and their ED_50_ values were 0.3±0.1 and 0.2±0.1 µM, respectively.

**Figure 2:**
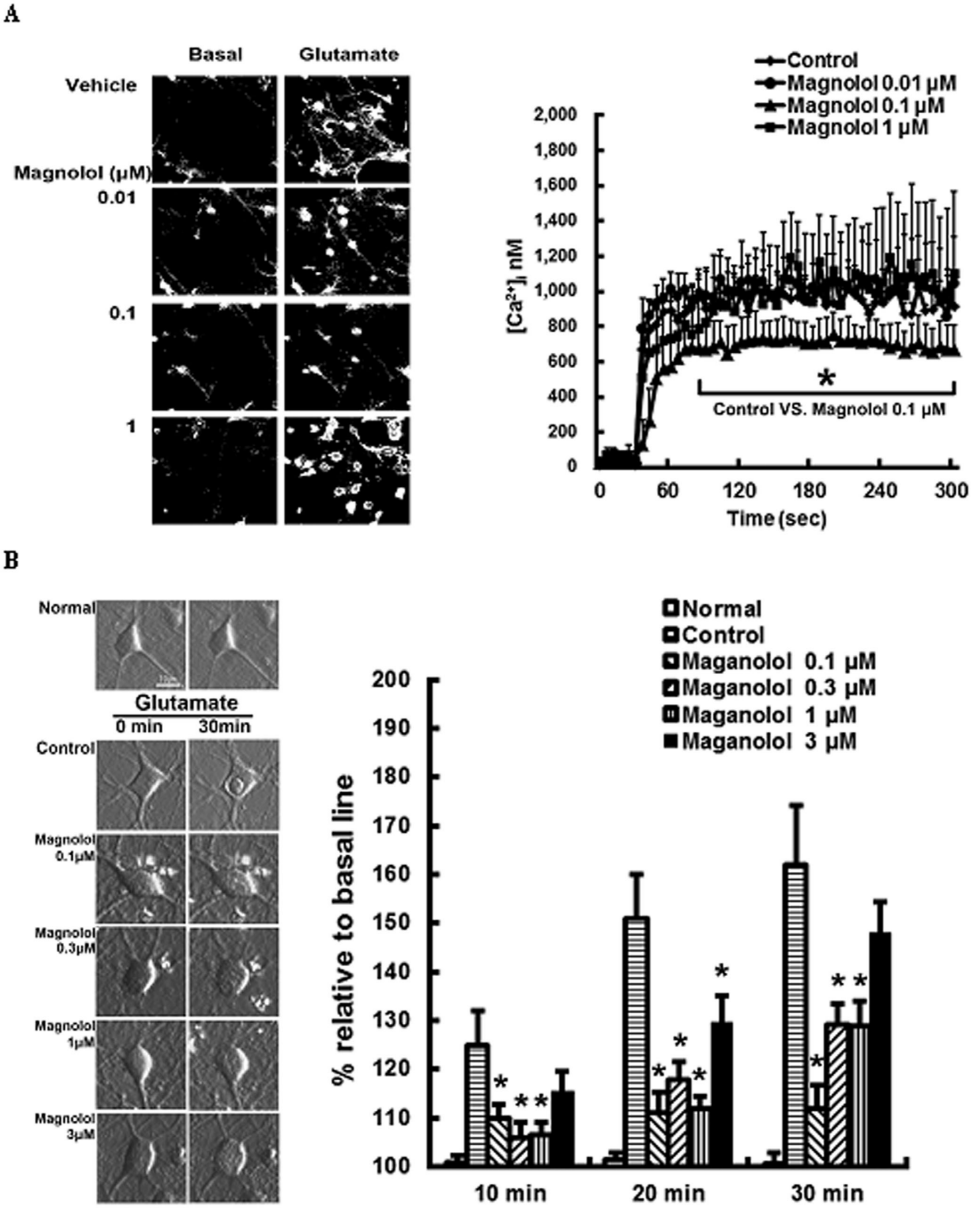
Magnolol attenuated glutamate-induced rises in the intracellular calcium, [Ca^2+^](i), inflow and cell swelling in cultured neurons. (A) Ratio image detection for [Ca^2+^](i) concentrations showed that magnolol at 0.1 µM, but not at 0.01 and 1 µM, effectively inhibited the rises of [Ca^2+^](i) induced by glutamate exposure. (B) Time-course differential interference contrast (DIC) photomicrographs of cultured neurons showed that magnolol at 0.1–1 µM attenuated the glutamate-induced cell swelling over time. *P<0.05 vs controls, and *n* = 6–7 per group.

At 10 *DIV* cultured neurons, the addition of glutamate (300 µM) induced abrupt rises of [Ca^2+^](i) levels up to ≈1000 nM. Magnolol, however, exhibited a hormetic inhibitory response. Only magnolol at 0.1 µM, but not at 0.01 and 1 µM, effectively inhibited the increased [Ca^2+^](i) inflow over time (*P<*0.05; Figs. 2A). On contrast, treatment with magnolol at 0.1–1 µM invariably attenuated the glutamate-induced neuronal cell swelling over time (*P<*0.05; Figs. 2B).

Nine animals (7.8%) died prior to completing the protocol following pMCAO and were excluded: 4 (8.2%) were in the vehicle-treated groups and 5 (5.4%) were in the magnolol-treated groups. Following the ischemic onset, the ipsilateral LCBP declined to 14–22% and 32–38% of the baseline data in the ischemic core and penumbral areas, respectively. The LCBP was not significantly different among experiment groups, and was independent of magnolol treatments (*P*>0.05; data not shown). The other physiological parameters were kept within normal limits and did not differ significantly among experiment groups, except that high-dose (200 mg/kg) magnolol-treated animals had arterial pCO_2_ retention along with reduced heart rate and arterial pH (Table 1). Ischemic animals invariably experienced spontaneous hyperthermia throughout the recovery period. Animals treated with magnolol at 200 mg/kg, but not at lower doses, had modest temperature reductions by ≈3°C, and this temperature-lowering effect remained effective up to 2 hrs post-insult (Table 2).

**Table 1:** Physiologic parameters before (preocclusion) and after (postocclusion) permanent middle cerebral artery occlusion (PMCAO) between animals pretreated with magnolol versus vehicle (PEG 400)-treated controls.

	n	pH	pCO_2_(mmHg)	pO_2_(mmHg)	Gluc(mg/dL)	Hct(%)	MABP(mmHg)	HR(beats/min)
Preocclusion								
Control	15	7.42±0.04	39.4±4.8	140.7±24.9	152±18	40.1±2.1	94±13	325±38
Magnolol-treated								
25 mg/kg	13	7.37±0.07	39.5±4.4	150.3±34.2	159±23	40.4±1.2	96±11	316±37
50 mg/kg	11	7.37±0.06	39.1±3.9	146.7±32.6	166±19	39.7±1.2	94±12	298±26
100 mg/kg	8	7.36±0.05	38.3±7.8	154.9±33.6	153±37	39.8±1.4	96±6	311±48
150 mg/kg	9	7.38±0.09	40.2±10.0	149.7±29.1	166±29	40.3±1.2	95±10	316±50
200 mg/kg	12	7.34±0.03*	46.3±10.2*	141.8±23.9	153±24	40.3±1.0	97±13	293±24*
Postocclusion								
Control	15	7.40±0.04	39.1±5.3	145.3±24.9	146±12	39.9±1.6	97±15	330±26
Magnolol-treated								
25 mg/kg	13	7.41±0.05	41.8±4.0	149.6±32.9	152±18	39.8±1.1	92±10	333±31
50 mg/kg	11	7.37±0.06	40.7±3.0	163.6±39.9	163±21	39.3±0.9	97±15.	322±28
100 mg/kg	8	7.38±0.07	41.8±6.3	153.5±33.0	148±36	38.9±1.4	96±12	333±40
150 mg/kg	9	7.38±0.08	40.9±9.2	15.4±22.6	159±29	39.3±1.2	97±6	332±35
200 mg/kg	12	7.37±0.09	44.6±10.3	140.8±28.1	143±30	39.5±1.0	95±8	304±14*

Physiologic data obtained from control and pre-treated animal groups are represented as the mean±standard deviation (SD). Hct – hematocrit; Gluc - blood glucose; MABP - mean arterial blood pressure; HR - heart rate; *n* – number of animals. All animals were maintained at 37±0.5°C. Paired Students’ *t* tests were used to evaluate the response to a change in conditions, and one-way Analysis of Variance (one-way ANOVA) with Dunnett’s *posthoc* comparison was used to evaluate differences between groups. The symbol * and **†** mean *P<*0.05, compared to preischemic and control data, respectively.

**Table 2:** The changes of core temperatures obtained after vehicle or magnolol treatment in rats subjected to permanent middle cerebral artery occlusion (PMCAO) in the study.

		*n*		Before PMCAO		10 min after PMCAO		1 h		2 h		4 h		24 h		
Pretreatment groups																
Control	15		36.8±0.1		36.8±0.2		38.0±0.1		38.1±0.1		38.2±0.3		38.0±0.3			
Magnolol																
25 mg/kg	13		36.8±0.3		36.7±0.3		37.9±0.3		38.0±0.3		38.2±0.4		38.1±0.3			
50 mg/kg	11		36.8±0.4		36.5±0.5		37.9±0.4		37.9±0.5		37.9±0.4		38.1±0.2			
100 mg/kg	8		36.9±0.4		36.7±0.5		37.8±0.4		37.8±0.5		37.8±0.5		38.0±0.3			
150 mg/kg	9		36.9±0.4		36.5±0.3		37.8±0.3		37.6±0.4		37.7±0.4		37.9±0.3			
200 mg/kg	12		36.0±0.2*		35.0±0.3*		35.1±0.3*		36.5±0.3*		37.8±0.3		37.7±0.2			

Data are represented as the mean±standard deviation (S.D.). *n* – number of animals.

* means *P*<0.05, compared to control data, respectively.

**Figure 3:**
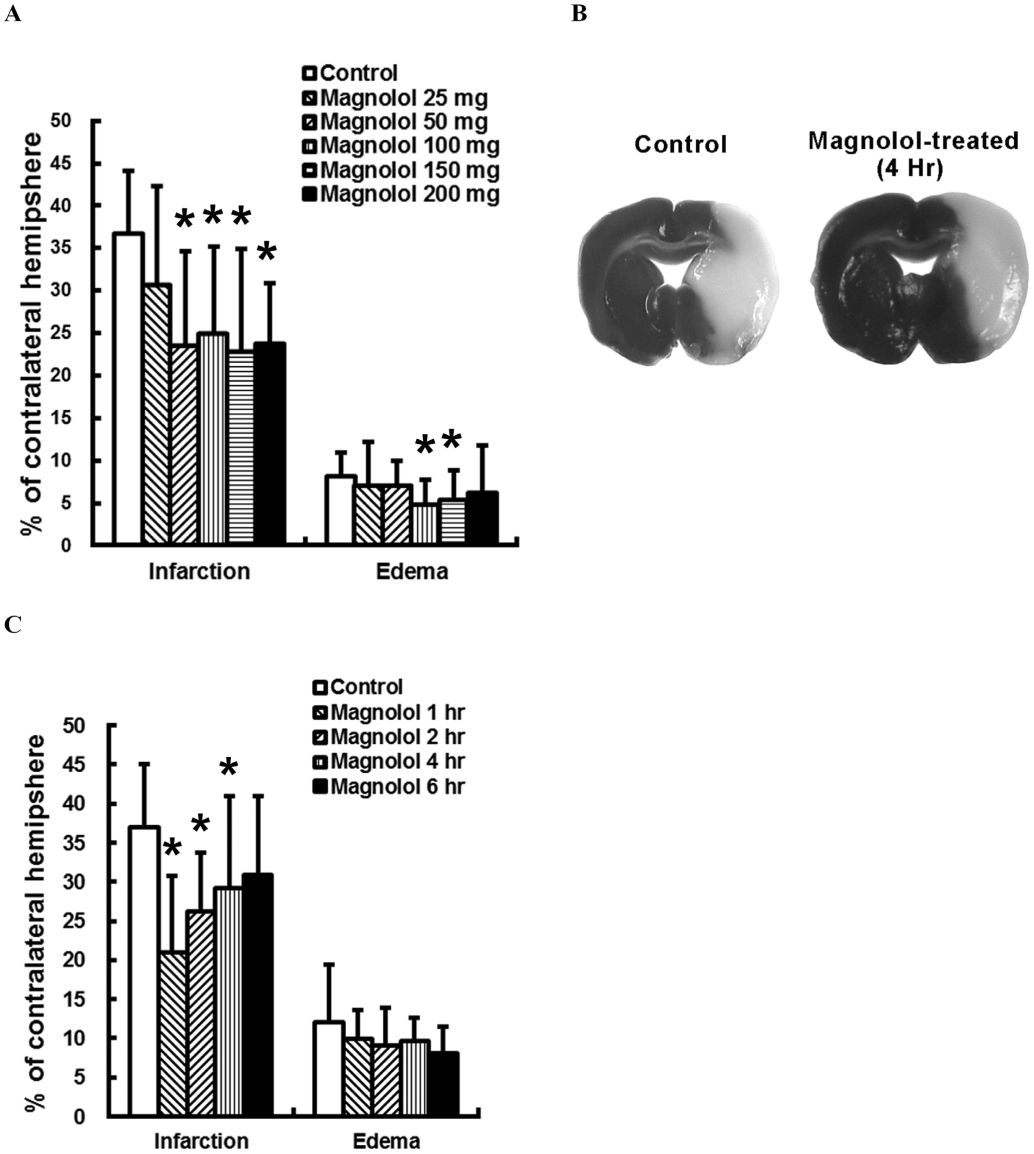
Magnolol reduced brain infarction in rats subjected to permanent middle cerebral artery (pMCAO) occlusion. Animals which were subjected to pMCAO and received an intraperitoneal injection of PEG 400 (*n* = 15) or magnolol, at 25 mg/kg (*n* = 13), 50 mg/kg (*n* = 11), 100 mg/kg (*n* = 8), 150 mg/kg (*n* = 9), or 200 mg/kg (*n* = 12), 1 hr before the ischemic insult. Infarct volumes were significantly reduced with magnolol treatment at 50–200 mg/kg (A), but not at 25 mg/kg. (B) The 2, 3, 5-Triphenyltetrazolium chloride (TTC)-stained coronal sections were from representative animals which received an intraperitoneal injection of PEG 400 or magnolol (100 mg/kg), at 4 hrs post-insult. Infarcts observed (pale region) involving the cerebral cortex and underlying striatum are substantially smaller in the magnolol-treated group. (C) Delayed treatment with magnolol (100 mg/kg) at 1 (*n* = 7), 2 (*n* = 7), or 4 hrs (*n* = 9), but not 6 hrs (*n* = 9), significantly reduced brain infarction, compared to controls (*n* = 30). The infarct volumes are expressed as a percentage of the contralateral hemisphere. *P<0.05 vs PEG 400-treated rats. *n*, number of animals.

Animals which were pre-treated with magnolol, at 50 mg/kg (n = 11), 100 mg/kg (n = 8), 150 mg/kg (n = 9), or 200 mg/kg (n = 12), but not at 25 mg/kg (n = 13), 1 hr before the ischemic onset, showed significant infarct size reductions (*P<*0.05) when compared with controls (n = 15). Infarction lesions were reduced by 30.9, 33.8, 37.8 and 35.3%, in animals treated with magnolol at 50 mg/kg, 100 mg/kg, 150 mg/kg, and 200 mg/kg, respectively (Fig. 3A). Animals treated with magnolol (100–200 mg/kg) also showed significantly improved sensory neurologic scores taken 22–24 hrs post-insult than did controls (*P<*0.05; Table 3). Additionally, significantly less body weight loss was observed in animals treated with magnolol (25–200 mg/kg), compared with controls (*P<*0.05; Table 3).

**Table 3:** Neurobehavioral scores and body weight loss obtained after permanent middle cerebral artery occlusion (pMCAO) in each pretreatment group in the study.

Neurobehavioral Scores				
	n	Motor	Sensory	BodyWeightLoss (g)
Pretreatment groups				
Control	15	2.0±0.3 (1.7–2.3)	3.5±0.4 (2.6–3.4)	36.5±8.3
Magnolol-treated				
25 mg/kg	13	2.0±0.2 (1.8–2.2)	3.0±0.7 (2.5–3.5)	28.0±6.0*
50 mg/kg	11	2.0±0.3 (1.7–2.3)	3.0±0.6 (2.4–3.6)	27.1±6.0*
100 mg/kg	8	2.0±0.2 (1.8–2.2)	2.0±0.5 (1.8–2.4)*	23.3±7.1*
150 mg/kg	9	2.0±0.2 (1.8–2.2)	2.0±0.5 (1.5–2.5)*	24.3±5.3*
200 mg/kg	12	2.0±0.2 (1.8–2.2)	2.0±0.4 (1.6–2.4)*	24.0±8.5*
Delayed treatment groups				
Control	30	2.0±0.2 (1.8–2.2)	4.0±0.2 (3.8–4.2)	38.7±7.9
Magnolol (100 mg/kg)				
1 hr	7	2.0±0.3 (1.7–2.3)	2.0±0.3* (1.7–2.3)	27.2±8.7*
2 hr	7	2.0±0.4 (1.6–2.4)	3.0±0.7* (2.3–3.7)	27.3±6.6*
4 hr	9	2.0±0.5 (1.5–2.5)	3.0±0.2* (2.8–4.0)	37.0±13.1
6 hr	9	2.0±0.3 (1.7–2.3)	3.5±0.7* (2.8–4.2)	41.8±13.5

Data are represented as the mean±standard deviation (S.D.). *n* – number of animals.

* means *P*<0.05, compared to control data, respectively.

In the delayed treatment paradigm, our results indicated that magnolol (100 mg/kg) resulted in significant infarct volume reductions when administrated within 4 hrs after the ischemic onset (*P*<0.05; Figs. 3B, C). Relative to controls (n = 30), infarction were reduced by 42.5, 28.5 and 20.6%, respectively, when magnolol was given at 1 (n = 7), 2 (n = 7), and 4 hrs (n = 9) post-insult (*P*<0.05; Figures 3C). Magnolol treated at 6 hrs post-insult (n = 9) did not significantly reduced brain infarction. However, delayed treatment with magnolol significantly improved sensory neurologic scores, even when administered up to 6 hrs post-insult (*P*<0.05), and effectively reduced post-ischemic body weight loss, when administered up to 2 hrs post-insult (*P*<0.05), but did not affect post-ischemic motor scores (*P>*0.05; Table 3). The physiological parameters were kept within normal limits and did not differ statistically between study and control animals (data not shown).

## Discussion

Our results indicated that magnolol (50–200 mg/kg) reduced infarct volumes and improved neurobehavioral outcomes in rats subjected to permanent focal cerebral ischemia. Additionally, we found that magnolol (100 mg/kg) was effective in reducing brain infarction and improving neurobehavioral outcomes even when administrated up to 4 hrs post-insult. Moreover, we demonstrated that magnolol not only effectively attenuated both glutamate- and NMDA-induced neurotoxicity, but also reduced the glutamate-induced increases in the [Ca^2+^](i) inflow and neuronal swelling. This neuroprotection cannot be accounted for by changes in glucose, hemodilution (as measured by blood hematocrit), or differences in mean arterial blood pressure, since these were not significantly different when compared between vehicle-injected and magnolol-treated animals. The changed physiologic parameters were decreases in arterial pH and heart rate, associated with a rise in pCO_2_, seen in the animals treated with magnolol at 200 mg/kg. These findings suggested that magnolol (200 mg/kg) might have induced a cardiopulmonary suppression, probably due to its centrally-acting muscular relaxant effect [12].

Exactly by which mechanisms in the glutamate-stimulated cultured neurons the dose-responsive regimen seen with magnolol for cell swelling inhibition was inconsistent with the “U-shaped” hormetic response observed for inhibiting the rises of [Ca^2+^](i) remains to be elucidated [18], [19]. Curiously, hormetic neuroprotective responses were also observed in the magnolol-treated stroke animals in which a low-dosing regimen was ineffective whereas high dosage (200 mg/kg) induced adverse effects along with a temperature-lowering action [12], [20], [21]. Thus, the *in vitro* dosing response might not represent the trend of dosing response observed *in vivo* [18]. It was very likely that magnolol actually had multiple mechanisms acted, independently or in combined, to exhibit neuroprotection observed here [12]–[17].

A therapeutic window of 4 hrs seen with magnolol in reducing brain infarction compares favorably with those of glutamate receptor antagonist and other anti-oxidant and radical-scavenging agents, but not as well as that reported with a calpain inhibitor [7], [8], [22]. Perhaps using multiple effective, smaller doses of magnolol, combined with an intravenous administration route, the therapeutic window may be extended and/or the degree of neuroprotection improved [8], [19]. Further studies are needed to determine whether magnolol can protect against reperfusion damage and late-onset ischemic insults following cerebral ischemia/reperfusion after a prolonged reperfusion period [23]. In additional, more mechanisms underlying neuroprotection observed here need to be elucidated.

In conclusion, magnolol protects against permanent focal cerebral ischemia with a therapeutic window up to 4 hrs post-insult. This neuroprotection may be partly mediated by its ability to attenuate the glutamate and NMDA-induced neurotoxicity.

## Materials and Methods

All procedures performed were approved by the Subcommittee on Research Animal Care of the University. All chemicals were purchased from Sigma-Aldrich Co. (St Louis, MO) unless otherwise indicated. Hank’s balanced salt solution (HBSS 10×, GIBCO, Grand Island, NY) was composed of (mM): glucose 55.56, KCl 53.33, NaCl 1379.31, KH_2_PO_4_ 0.44 and Na_2_PO_4_ 3.36; pH 7.1. Magnolol (Wako Pure Chemical Industries, Ltd., Osaka, Japan) was dissolved in PEG 400 or dimethylsulfoxide (DMSO).

### Neuronal Cultures and Cytotoxicity Assay

According the method described previously [18], cultured neurons were obtained from cerebral cortices of 1-day-old Sprague-Dawley rats. Cytotoxicity was determined at 24 hrs after treatment by using a LDH assay kit (Promega, Madison, WI) [18], [24], [25]. Experiments were undertaken on cultured neurons between 10 and 14 days *in vitro* (DIV). Neurons were incubated magnolol (0–300 µM) or vehicle (0.1% DMSO). The LD_50_ value was defined as the concentration of compound required to induce 50% of cell deaths in 24 hrs at 37°C.

### Glutamate- and N-methyl-D-aspartate (NMDA)-induced Cell Cytotoxicity

Cultured neurons were pre-treated with magnolol (0.1–1 µM) or vehicle (0.1% DMSO) for 30 min and, then, were exposed to glutamate (300 µM) or NMDA (100 µM) for 24 hrs. The ED_50_ value was defined as the concentration of compound required to reduce 50% of cell deaths of controls in 24 hrs at 37°C.

### Intracellular Ca^2+^ Measurement

The level of [Ca^2+^](i) were measured on a single cell fluorimeter [26], [27]. Briefly, neuronal cultures were incubated with 3 µM fura 2-acetoxymethylester (Fura-2 AM) and 10 µM ionomycin in a standard buffer (composition in mM: NaCl, 140; KCl, 3.5; KH_2_PO_4_, 0.4; Na_2_HPO_4_, 1.25; CaCl_2_, 2.2; MgSO_4_, 2; glucose, 10; HEPES, 10, pH 7.3) for 30 min, followed by incubation in dye-free standard buffer for 30 min and, then, the addition of vehicle or magnolol (0.01, 0.1, or 1 µM) for 20 min and the exposure of glutamate (300 µM). During experiments, standard buffer was replaced by low Mg^2+^ saline (composition in mM: NaCl, 140; KCl, 3.5; KH_2_PO_4_, 0.4; Na_2_HPO_4_, 1.25; CaCl_2_, 2.2; MgSO_4_, 0.03; glucose, 10; HEPES, 10, pH 7.3). The glass coverslip was placed into the stage chamber of an Olympus IX71 inverted microscope, equipped with a 75 W xenon illumination system, a cooled charge-couple device (CCD) camera (300T-RC; Dage-MTI, Michigan City, IN) coupled to an image intensifier (Gen II S-25 image intensifier; Dage-MTI), a Lambda 10-2 filter-wheel and shutter (Sutter Instruments, Novato, CA) and a computerized image analyzer (MCID Elite, Imaging Research Inc., St. Catherines, Ontario, Canada). The cells were alternatively illuminated with the light of 340 and 380 nm wavelengths and the emitted light was passed through a 510 nm barrier filter. The 340 and 380 nm images were captured at 6 second intervals and the ratio signals (340 nm excited image/380 nm excited image) were processed and examined for real changes in [Ca^2+^](i)_._ Approximately 10 neurons in each microscopic field were individually measured. The [Ca^2+^](i) level was calculated by using the equation: [Ca^2+^](i)  =  Kd×(Fo/Fs)×[(R−Rmin)/(Rmax−R)] where Kd is the dissociation constant for fura -2 in the cytosol (225 nM), and Fo/Fs is the fluorescence emitted at 380 nm excitation at minimum Ca^2+^ level divided by the same emission fluorescence at the fura-saturated concentration [28]. R is the ratio fluorescence intensity recorded at 340 and 380 nm, and Rmin and Rmax are the rations of 340/380 nm fluorescence intensity recorded at minimum Ca^2+^ and the fura-saturated Ca^2+^ concentrations, respectively. We used the Fura-2 Calcium Imaging Calibration Kit (F-6774; Invitrogen Molecular Probes, Eugene, OR) to detect the Kd level under conditions. Measurements of Fo and Rmin were performed in nominally Ca^2+^-free isotonic solution containing 10 mM EGTA. Cells were then superfused with isotonic solution containing 1 µM thapsigargin, 10 µM ionomycin and 10 mM Ca^2+^ to evaluate Fs and Rmax.

### Cell Swelling Measurements

The glutamate (300 µM)-induced neuronal morphologic changes were measured by time-lapse imaging techniques in a microscope equipped with a thermo-controllable heating stage, differential interference contrast (DIC) lens and an image analyzer (MCID Elite) by the method described previously [29], [30]. DIC images of pyramid-shaped neurons were measured and compared over time. Three randomly selected fields were counted and averaged per culture (approximately 12 to 15 neurons per culture). Data are expressed as a percentage relative to the baseline values.

### Animal Preparation, Anesthesia, and Monitoring

Male Sprague-Dawley rats, weighting 220–270 g, were supplied by the University Laboratory Animal Center, and were allowed free access to food and water before and after surgery. Animals were anesthetized with 1–2% halothane in 70% N_2_O/30% O_2_. During surgery, body temperature was maintained at 37±0.5°C using a thermostatically controlled heating blanket and rectal probe (Harvard Apparatus, South Natick, MA). The right femoral artery was cannulated for measuring arterial blood gases, glucose, hematocrit and blood pressure [8], [18], [24], [25].

### Experimental Model

Focal cerebral ischemia was employed by permanent occlusion of the proximal right middle cerebral artery (pMCAO) with a 4-0 nylon suture occluder, as described previously [8], [18], [24], [25]. Successful MCA occlusion was ensured by a sharp decrease of local cortical blood perfusion (LCBP) to about 20% of baseline as determined by Laser-Doppler flowmetry (LDF, Laserflo BMP^2^, Vasamedics, St. Paul, MN) [8], [9], [19], [23], [31].

### Drug Administration and Grouping of Animals

In the first series of experiments, animals were received either magnolol (25 mg/kg, 50 mg/kg, 100 mg/kg, 150 mg/kg, or 200 mg/kg, i.p.; n = 58) or vehicle (the same volume of PEG 400, i.p.; n = 16), 1 hr pre-insult, to test the neuroprotective dose response. An additional set of rats, received magnolol (100 mg/kg, i.p.; n = 34) or vehicle (PEG 400, i.p.; n = 33) at 1, 2, 4 or 6 hrs post-insult, was used to evaluate the therapeutic window of opportunity.

### Neurobehavioral Testing

Neurologic and body weight measurements were conducted by an investigator unaware of treatment protocol at 24 hrs post-insult [8], [18], [24], [25], [32]. Five categories of motor neurologic findings were scored: 0, no observable deficit; 1, forelimb flexion; 2, forelimb flexion and decreased resistance to lateral push; 3, forelimb flexion, decreased resistance to lateral push and unilateral circling; 4, forelimb flexion, unable or difficult to ambulate. The affected forelimb also received forward and sideways visual placing tests which were scored as follows: 0, complete immediate placing; 1, incomplete and/or delayed placing (<2 seconds); 2, absence of placing.

### Animal Sacrifice and Quantification of Ischemic Damage

Sacrifice was performed at 24 hrs post-insult by decapitation under anesthesia. The brain was cut into 2 mm coronal sections using a rat brain matrix (RBM 4000 C, ASI Instrument, Inc., Warren, MI) and stained according to standard 2, 3, 5-triphenyltetrazolium chloride (TTC) method [8], [24]. Briefly, the brain was cut into 2 mm coronal sections using a rat brain matrix (RBM 4000 C, ASI Instrument, Inc., Warren, MI) and stained according to the standard 2, 3, 5-triphenyltetrazolium chloride (TTC) method [8], [19], [24]. A total of 7 brain sections were traced and measured using a computerized image analyzer (MCID Elite). The calculated infarction areas were then compiled to obtain the infarct volumes per brain (in mm^3^). Brain Infarct volumes were expressed as a percentage of the contralateral hemisphere volume [8], [24].

### Statistical Analysis

All data were expressed as the mean±standard deviation (S.D.). Paired Students’ *t* test was used to evaluate the response to a change in conditions, and unpaired Students’ *t* test/one-way analysis of variance (one-way ANOVA) with Fisher’s protected least significant difference (LSD) *posthoc* comparison was used to evaluate differences between groups. Neurobehavioral scores were analyzed by the Kruskal-Wallis/Mann-Whitney *U* test. *P*<0.05 was selected for statistical significance.

## References

[1] Choi DW (1998). Glutamate neurotoxicity and diseases of the nervous system.. Neuron.

[2] Papadia S, Stevenson P, Hardingham NR, Bading H, Hardingham GE (2005). Nuclear Ca^2+^ and the cAMP response element-binding protein family mediate a late phase of activity-dependent neuroprotection.. J Neurosci.

[3] Papadia S, Soriano FX, Leveille F, Martel MA, Dakin KA (2008). Synaptic NMDA receptor activity boosts intrinsic antioxidant defenses.. Nat Neurosci.

[4] Zhang M, Xu JT, Zhu X, Wang Z, Zhao X (2010). Postsynaptic density-93 deficiency protects cultured cortical neurons from N-methyl-D-aspartate receptor-triggered neurotoxicity.. Neuroscience.

[5] Fan J, Vasuta OC, Zhang LY, Wang L, George A (2010). N-methyl-D-aspartate receptor subunit- and neuronal-type dependence of excitotoxic signaling through post-synaptic density 95.. J Neurochem.

[6] Clemens JA (2000). Cerebral ischemia: gene activation, neuronal injury, and the protective role of antioxidants.. Free Radic Biol Med.

[7] Markgraf CG, Velayo NL, Johnson MP, McCarty DR, Medhi S (1998). Six-hour window of opportunity for calpain inhibition in focal cerebral ischemia in rats.. Stroke.

[8] Lee EJ, Chen HY, Wu TS, Chen TY, Ayoub IA (2002). Acute administration of Ginkgo biloba extract (EGb 761) affords neuroprotection against permanent and transient focal cerebral ischemia in Sprague-Dawley rats.. J Neurosci Res.

[9] Lee EJ, Chen HY, Lee MY, Chen TY, Hsu YS (2005). Cinnamophilin reduces oxidative damage and protects against transient focal cerebral ischemia in mice.. Free Radic Biol Med.

[10] Ritz MF, Curin Y, Mendelowitsch A, Andriantsitohaina R (2008). Acute treatment with red wine polyphenols protects from ischemia-induced excitotoxicity, energy failure and oxidative stress in rats.. Brain Res.

[11] Lin SP, Tsai SY, Lee Chao PD, Chen YC, Hou YC (2011). Pharmacokinetics, bioavailability, and tissue distribution of magnolol following single and repeated dosing of magnolol to rats.. Planta Med.

[12] Watanebe K, Watanabe H, Goto Y, Yamaguchi M, Yamamoto N (1983). Pharmacological properties of magnolol and honokiol extracted from Magnolia officinalis: central depressant effects.. Planta Med.

[13] Chen HY, Hung YC, Lee EJ, Chen TY, Chuang IC (2009). The protective efficacy of magnolol in hind limb ischemia-reperfusion injury.. Phytomedicine.

[14] Chang CP, Hsu YC, Lin MT (2003). Magnolol protects against cerebral ischaemic injury of rat heatstroke.. Clin Exp Pharmacol Physiol.

[15] Lee MM, Hseih MT, Kuo JS, Yeh FT, Huang HM (1998). Magnolol protects cortical neuronal cells from chemical hypoxia in rats.. NeuroReport.

[16] Lin YR, Chen HH, Ko CH, Chan MH (2006). Neuroprotective activity of honokiol and magnolol in cerebellar granule cell damage.. Eur J Pharmacol.

[17] Teng CM, Yu SM, Chen CC, Huang YL, Huang TF (1990). EDRF-release and Ca+^2^-channel blockade by magnolol, an antiplatelet agent isolated from Chinese herb Magnolia officinalis, in rat thoracic aorta.. Life Sci.

[18] Tai SH, Hung YC, Lee EJ, Lee AC, Chen TY (2011). Melatonin protects against transient focal cerebral ischemia in both reproductively active and estrogen-deficient female rats: the impact of circulating estrogen on its hormetic dose-response.. J Pineal Res.

[19] Lee EJ, Hung YC, Chen HY, Wu TS, Chen TY (2009). Delayed treatment with carboxy-PTIO permits a 4-hour therapeutic window of opportunity and prevents against ischemia-induced energy depletion following permanent focal cerebral ischemia in mice.. Neurochem Res.

[20] Hsieh MT, Chueh FY, Lin MT (1988). Magnolol decreases body temperature by reducing 5-hydroxytryptamine release in the rat hypothalamus.. Clin Exp Pharmacol Physiol.

[21] Yanamoto H, Nagata I, Niitsu Y, Zhang Z, Xue JH (2001). Prolonged mild hypothermia therapy protects the brain against permanent focal ischemia.. Stroke.

[22] Hatfield RH, Gill R, Brazell C (1992). The dose-response relationship and therapeutic window for dizocilpine (MK-801) in a rat focal ischaemia model.. Eur J Pharmacol.

[23] Chen TY, Tai SH, Lee EJ, Huang CC, Lee AC (2011). Cinnamophilin offers prolonged neuroprotection against gray and white matter damage and improves functional and electrophysiological outcomes following transient focal cerebral ischemia.. Crit Care Med.

[24] Lee EJ, Chen HY, Hung YC, Chen TY, Lee MY (2009). Therapeutic window for cinnamophilin following oxygen-glucose deprivation and transient focal cerebral ischemia.. Exp Neurol.

[25] Chen TY, Lin MH, Lee WT, Huang SY, Chen YH (2012). Nicotinamide inhibits nuclear factor-kappa B translocation after transient focal cerebral ischemia.. Crit Care Med.

[26] Williams DA, Fay FS (1990). Intracellular calibration of the fluorescent calcium indicator Fura-2.. Cell Calcium.

[27] Hedrick MS, Fahlman CS, Bickler PE (2005). Intracellular calcium and survival of tadpole forebrain cells in anoxia.. J Exp Biol.

[28] Grynkiewicz G, Poenie M, Tsien RY (1985). A new generation of Ca2+ indicators with greatly improved fluorescence properties.. J Biol Chem.

[29] Sun DA, Sombati S, DeLorenzo RJ (2001). Glutamate injury-induced epileptogenesis in hippocampal neurons: an in vitro model of stroke-induced “epilepsy”. Stroke.

[30] DeLorenzo RJ, Sun DA, Blair RE, Sombati S (2007). An in vitro model of stroke-induced epilepsy: elucidation of the roles of glutamate and calcium in the induction and maintenance of stroke-induced epileptogenesis.. Int Rev Neurobiol.

[31] Chen HY, Hung YC, Chen TY, Huang SY, Wang YH (2009). Melatonin improves presynaptic protein, SNAP-25, expression and dendritic spine density and enhances functional and electrophysiological recovery following transient focal cerebral ischemia in rats.. J Pineal Res.

[32] Tai SH, Chen HY, Lee EJ, Chen TY, Lin HW (2010). Melatonin inhibits postischemic matrix metalloproteinase-9 (MMP-9) activation via dual modulation of plasminogen/plasmin system and endogenous MMP inhibitor in mice subjected to transient focal cerebral ischemia.. J Pineal Res.

